# Multi-organ Radiomics-Based Prediction of Future Remnant Liver Hypertrophy Following Portal Vein Embolization

**DOI:** 10.1245/s10434-023-14241-5

**Published:** 2023-09-05

**Authors:** Mirjam Gerwing, Philipp Schindler, Shadi Katou, Michael Köhler, Anna Christina Stamm, Vanessa Franziska Schmidt, Walter Heindel, Benjamin Struecker, Haluk Morgul, Andreas Pascher, Moritz Wildgruber, Max Masthoff

**Affiliations:** 1 Clinic for Radiology, University Hospital Münster, Münster, Germany; 2 Department for General, Visceral and Transplant Surgery, University Hospital Münster, Münster, Germany; 3 Department for Radiology, University Hospital, LMU Munich, Munich, Germany

**Keywords:** Radiomics, Future remnant liver hypertrophy, Portal vein embolization, Outcome prediction

## Abstract

**Background:**

Portal vein embolization (PVE) is used to induce remnant liver hypertrophy prior to major hepatectomy. The purpose of this study was to evaluate the predictive value of baseline computed tomography (CT) data for future remnant liver (FRL) hypertrophy after PVE.

**Methods:**

In this retrospective study, all consecutive patients undergoing right-sided PVE with or without hepatic vein embolization between 2018 and 2021 were included. CT volumetry was performed before and after PVE to assess standardized FRL volume (sFRLV). Radiomic features were extracted from baseline CT after segmenting liver (without tumor), spleen and bone marrow. For selecting features that allow classification of response (hypertrophy ≥ 1.33), a stepwise dimension reduction was performed. Logistic regression models were fitted and selected features were tested for their predictive value. Decision curve analysis was performed on the test dataset.

**Results:**

A total of 53 patients with liver tumor were included in this study. sFRLV increased significantly after PVE, with a mean hypertrophy of FRL of 1.5 ± 0.3-fold. sFRLV hypertrophy ≥ 1.33 was reached in 35 (66%) patients. Three independent radiomic features, i.e. liver-, spleen- and bone marrow-associated, differentiated well between responders and non-responders. A logistic regression model revealed the highest accuracy (area under the curve 0.875) for the prediction of response, with sensitivity of 1.0 and specificity of 0.5. Decision curve analysis revealed a positive net benefit when applying the model.

**Conclusions:**

This proof-of-concept study provides first evidence of a potential predictive value of baseline multi-organ radiomics CT data for FRL hypertrophy after PVE.

**Supplementary Information:**

The online version contains supplementary material available at 10.1245/s10434-023-14241-5.

In advanced liver cancer, major surgery is commonly limited by tumor location and remaining future remnant liver volume (FRLV).^1,2^ Insufficient FRLV before surgery has been shown to be a major risk factor of postoperative liver failure associated with high mortality.^3,4^ To minimize the exclusion of patients from major surgery and risk of postoperative liver failure, several techniques, such as associated liver partition and portal vein ligation for staged hepatectomy (ALPPS) or portal vein embolization (PVE) alone or with additional hepatic vein embolization (HVE), have been introduced to increase FRLV prior to liver surgery.^5,6^ PVE, as a minimally invasive technique, has proved to efficiently induce hypertrophy of FRLV securing postoperative liver function.^6–9^ Although distinct cut-off values are still discussed in the current literature, an FRLV of > 20–25% for patients with a normal liver, > 30% for patients with steatosis/steatohepatitis, and up to > 40% for patients with liver cirrhosis will commonly be achieved.^1,10,11^ However, there is still a high rate of patients failing to proceed to surgery after PVE due to insufficient hypertrophy.^12^ To *a priori* identify non-responders of PVE in advance can preserve patients from a procedure and its associated short- or long-term risks. However, although some studies found predictive parameters for FRLV hypertrophy after PVE, there is no reliable parameter or gold standard to predict sufficient FRLV hypertrophy prior to PVE. Meanwhile, there have been studies assessing and predicting liver function and risk of postoperative liver failure based on imaging by using radiomic approaches.^13–15^ Radiomics is a promising quantitative approach that extracts high amounts of specific radiomic features, which are distinctive, standardized and high-dimensional image characteristics beyond those observables by the naked eye, from medical imaging data. The derived radiomic prediction models may characterize defined conditions of the displayed pathology or predict therapy outcome and thus support clinical decision making towards personalized medicine.^16–18^ This study aimed to evaluate the feasibility of radiomic features extracted from baseline abdominal computed tomography (CT) to predict FRLV hypertrophy after PVE.

## Methods

### Study Design

This study was carried out as a retrospective, single-center, observational trial in a tertiary care academic liver center. The trial was conducted in accordance with both the Declaration of Helsinki and the Declaration of Istanbul, and was approved by the local Ethics Committee of Westfalen-Lippe, Münster, Germany (ID 2019-636-f-S). Due to the retrospective character of this study, the informed consent of patients was waived. This study was reported in line with the Strengthening the Reporting of Observational Studies in Epidemiology (STROBE) criteria.

### Patient Selection and Data Collection

All consecutive patients undergoing right-sided PVE with or without additional hepatic vein embolization (HVE) between 2018 and 2021 were included. All patients undergoing PVE had the approval of the interdisciplinary gastrointestinal tumor board.

All patient and procedural data as well as follow-up data were retrospectively acquired from the electronic patient records as well as from the Picture Archiving and Communications System (PACS).

### Procedure Details of Portal Vein Embolization (PVE)

**Fig. 1:**
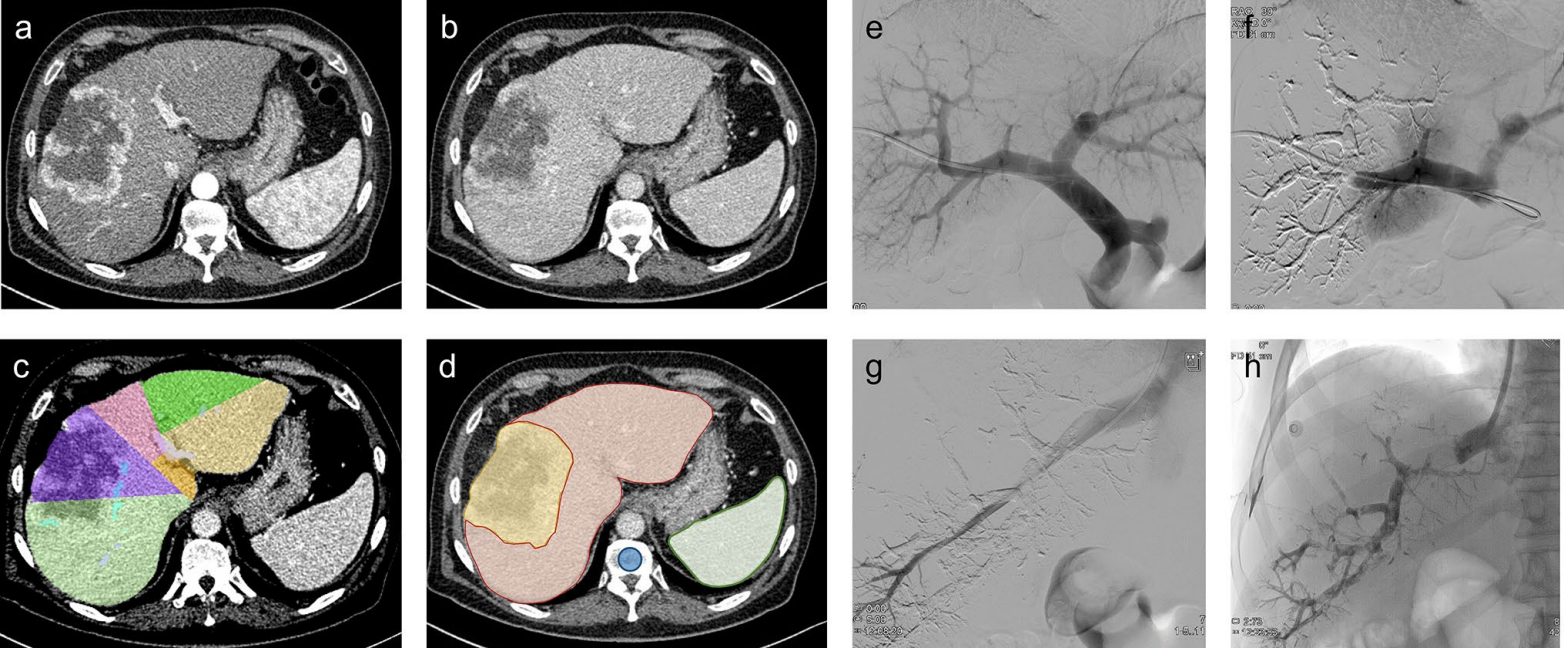
Representative CT imaging of a cholangiocarcinoma in the **a** arterial and **b** portal venous phases. **c** CT volumetry of the liver was performed to calculate FRLV and sFRLV. **d** Three-dimensional segmentation of the liver (without the tumor) and the spleen as well as ROI placement in the L1 vertebra for radiomics analysis was performed by two experienced radiologists in the portal venous phase of baseline CT. **e**–**h** Representative peri-interventional fluoroscopic images of a portal and hepatic vein embolization. *CT* Computed tomography, *FRLV* Future remnant liver volume, *sFRLV* Standardized future remnant liver volume, *ROI* Region of interest

Interventional procedures were performed as previously described.^19^ Briefly, a right-sided segmental portal vein branch was punctured with a 21G Chiba needle (Boston Scientific, Miami, FL, USA) under ultrasound guidance, followed by introduction of a 5F sheath (Accustick, Boston Scientific). A 6F 45 cm sheath was then introduced via an ultra-stiff .038 inch guidewire. After portography, right-sided PVE was performed using a coaxial system with a 5F S1 configurated diagnostic catheter and a 2.7F microcatheter. A mixture of 1 mL of n-butyl-cyanoacrylate (Glubran II; GEM, Viareggio, Italy) as an embolic agent and 5–6 mL of iodized oil (Lipiodol; Guerbet, Aulnay-sous-Bois, France) was injected via the microcatheter after flushing with 10% glucose. Whenever a trisectionectomy was planned, the segment IV portal vein branch was additionally occluded in the same technique, if safely feasible. Finally, the puncture tract was occluded with a mixture of 1 mL of n-butyl-cyanoacrylate (Glubran II; GEM) as an embolic agent and 2–3 mL of iodized oil (Lipiodol; Guerbet). In the case of additional HVE, the right jugular vein was subsequently punctured under sonographic guidance. After introduction of a 7–10F sheath, the right hepatic vein was occluded by an 18–22 mm Amplatzer Vascular Plug II or IV (St Jude Medical, Saint Paul, MN, USA). Whenever a trisectionectomy was planned, the middle hepatic vein was additionally occluded in the same technique. Exemplary CT and peri-interventional fluoroscopic images of PVE and HVE are shown in Fig. 1.

### Computed Tomography Volumetry

CT volumetry was performed prior to baseline and at 2.8 ± 0.9 weeks (range 2–5 weeks) after an interventional procedure to evaluate hypertrophy of the left liver (Fig. 1c) using venous phase axial slices with an IntelliSpace Portal station (Philips, Best, The Netherlands) CT viewer software to assess FRLV.^20^ FRLV was defined as segments II–III in all patients to ensure homogeneity of the study cohort. Furthermore, literature-based standardized FRLV (sFRLV),^19,21^ where FRLV is calculated in relation to total liver volume based on body surface area (BSA), was calculated as: sFRLV [%] = FRLV / (−794.41 + 1267.28 × BSA) × 100 with BSA = [weight (kg) × height (cm)/3600].^22^ This method has the advantage of avoiding errors due to the subtraction of multiple tumors, the inclusion of non-functional liver with dilated ducts in the measurements, and the use of a different denominator between pre- and post-PVE total liver volume.^23^ This study aimed to predict actual liver hypertrophy, therefore response classification was based on sFRLV increase, assessed by a ratio of the degree of hypertrophy after PVE compared with prior PVE. Therefore, sufficient response to interventional procedure (FRL hypertrophy) was defined as an increase in sFRLV by ≥ 1.33 after PVE compared with baseline, as similarly established in the literature.^24,25^

### Image Segmentation and Feature Extraction

Two experienced radiologists, blinded for clinical and interventional data, reviewed the baseline contrast-enhanced CT images of the abdomen. The readers chose liver and spleen parenchyma as well as bone marrow, with these being defined as possible influencing factors for liver hypertrophy after PVE. Thereafter, the readers independently segmented the entire liver (without tumor) and spleen volume, as well as a 1 cm^3^ volume-of-interest (VOI) of the first lumbar vertebral bone marrow, each as a separate label map. Liver-, splenic-, and bone marrow-specific radiomic features from labeled CT data were extracted twice, each by the same independent readers, for interobserver analysis, and included 162 first-order logic features and 216 grey level co-occurrence matrix (GLCM) features. These features are used to quantify shape (e.g., compactness, sphericity), intensity (e.g., histogram statistics of the mean, standard deviation, and median), and texture matrices, including the GLCM, where the differences represent the heterogeneity of the tissue. Image analysis and feature extraction was performed by using a freely available software package (3D slicer, version 4.11.2).

### Feature Selection and Model Analysis

Feature selection and dimension reduction were necessary as the number of radiomic features (*n* = 378) exceeded the number of patients (*n* = 53). Reproducibility of the extracted features between the two readers was assessed by calculating the concordance correlation coefficient for each of the features as a measure of intraclass correlation. Features with a coefficient between 0.8 and 1 were classified as ‘excellent’ and were included in further analysis. Using z-score standardization, all feature values were normalized to a range between 0 and 1, which improves comparability. The normalized dataset was randomly subdivided into a balanced training and test dataset (70/30 ratio). Further feature reduction was performed only on the training dataset using a Boruta machine-learning algorithm, which applies a random forest algorithm by performing a top-down search for relevant features. Irrelevant features were progressively eliminated to stabilize the model after comparison of the original attributes’ importance to importance achievable at random.^26^ Subsequently, a correlation matrix was calculated since there was no relevant gain in information in closely correlated features. A radiomics-based model was fitted on the test dataset based on a radiomics signature consisting of reliable radiomic features that allow classification of response (sFRLV hypertrophy by ≥ 1.33) using multivariate logistic regression. The discriminatory efficacy of the features was quantified by calculating the area under the curve (AUC) using the receiver operating characteristic (ROC) by applying a model-derived threshold at the highest sensitivity.

Decision curve analysis was performed on the test dataset to evaluate the clinical benefit of the prediction model. Decision curve analysis consisted of graphically showing the so-called ‘net benefit’ in function of the threshold probability.^27,28^ The preferences of patients and the attending physicians for performing PVE are accounted for by using a metric called threshold probability (*p*_t_).^29^ The rationale is that patients with a predicted probability of hypertrophy after PVE (*p*_p_) > (*p*_t_) are judged as positive and will be assigned to PVE; otherwise, those with (*p*_p_) < (*p*_t_) are judged negative and will not be treated. To compare two models at a predefined range of threshold probabilities, the concept of decision curve analysis is to calculate the net benefit (for the treated) using the following equation:

$${\text{net}}\;{\text{benefit}} = \frac{{{\text{true}}\;{\text{positives}}}}{{{\text{all}}\;{\text{patients}}}} - \frac{{{\text{false}}\;{\text{positives}}}}{{{\text{all}}\;{\text{patients}}}} \left( {\frac{{p_{t} }}{{1 - p_{t} }}} \right)$$

here the radiomics-based prediction model is compared with two default strategies: (1) assume that all patients test positive and therefore treat everyone; or (2) assume that all patients test negative and offer treatment to no-one. The radiomics-based model is said to be superior to the treat all or treat none strategies at the chosen threshold probability (*p*_t_) if its net benefit surpasses the net benefit of the other model for that value of (*p*_t_).

**Fig. 2:**
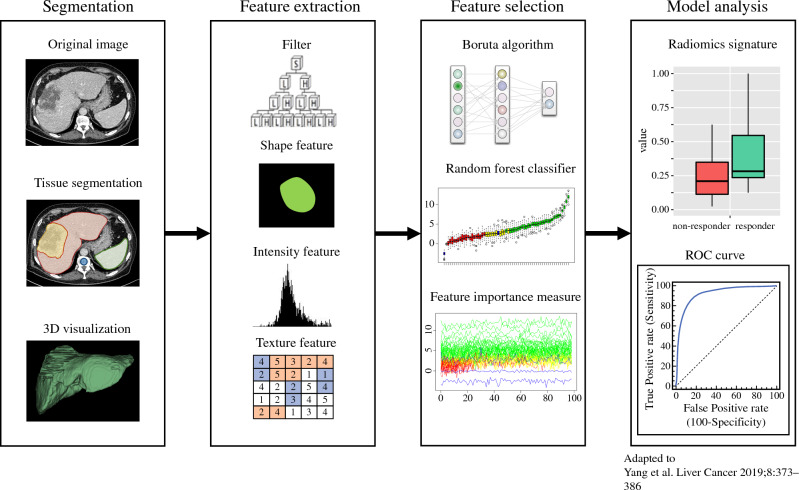
Scheme of radiomic workflow. After 3D segmentation of the liver (without the tumor), the spleen and the L1 vertebra representing the bone marrow, radiomic features, including shape, intensity and texture, were extracted after wavelet transform of the images. Feature reduction was performed with a random forest algorithm. Afterwards, radiomics signature was built with the logistic regression model and an ROC curve plotted. Scheme modified from Yang et al.^61^ *3D* Three-dimensional, *ROC* Receiver operating characteristics

The radiomics workflow is illustrated in Fig. 2. Radiomic feature selection and model analysis was performed by using an open-source software package (R/R studio, version 4.0.5; The R Foundation for Statistical Computing, Vienna, Austria).

### Statistical Analysis

Data are shown as mean and standard deviation or median and range, as appropriate. The paired Student’s *t*-test was used for comparison of related longitudinal volumetry data; in the case of non-related data, the unpaired Student’s* t*-test was used. Linear regression or binary logistic regression was performed for analysis of baseline characteristics correlating with higher FRL hypertrophy (sFRL ratio) or with response (sFRL hypertrophy of ≥ 1.33), respectively. A *p*-value < 0.05 was considered statistically significant. Statistical analysis was performed using Graph Pad Prism 5 (GraphPad Software, San Diego, CA, USA).

## Results

### Patient Characteristics

A total of 53 consecutive patients (21 females, 32 males), with a mean age of 64 ± 10.5 years (range 22–82 years), receiving PVE prior to major hepatectomy were included in this study. Nineteen patients (35.8%) received simultaneous HVE in addition to PVE, additional segment IV embolization was performed in 9 (17.0%) patients, and 4 (7.5%) patients received HVE and segment IV embolization. Patients had various entities of primary or secondary liver cancer, such as perihilar cholangiocarcinoma (*n* = 14), intrahepatic cholangiocarcinoma (*n* = 8), gall bladder carcinoma (*n* = 5), hepatocellular carcinoma (*n* = 7), colorectal cancer (*n* = 14), non-seminomatous germ cell tumors (*n* = 1), uveal malignant melanoma (*n* = 1), liver adenoma (*n* = 1), immunoglobulin (Ig) G4-associated mass (*n* = 1, initially suspected cholangiocarcinoma), and solitary fibrous tumor (*n* = 1). Nineteen patients (35.8%) in the cohort had known liver steatosis, 16 (30.1%) had liver fibrosis, and 2 (3.8%) had liver cirrhosis. Eighteen patients (34.0%) had a history of chemotherapy due to underlying primary or secondary liver cancer prior to PVE. Baseline liver function tests prior to PVE were 0.9 ± 1.1 mg/dL for bilirubin, 59.7 ± 47.9 U/L for glutamic oxaloacetic transaminase (GOT), 66.0 ± 63.9 U/L for glutamic pyruvic transaminase (GPT), and 372.0 ± 384.3 U/L for gamma-glutamyl transferase (GGT). Mean body mass index (BMI) was 26.8 ± 5.2 kg/m^2^. Detailed patient characteristics are reported in Table 1.

**Table 1:** Patient characteristics

Parameter	*n* (%)	*p* value^b^ sFRLV hypertrophy	*p* value^c^ sFRLV hypertrophy ≥ 1.33
All patients	53		
Age, years [median (range)]	64 (22–82)	> 0.05	> 0.05
Sex		0.045	> 0.05
Male	32 (60.38)		
Female	21 (39.62)		
Primary tumor		> 0.05	> 0.05
HCC	7 (13.21)		
CrC	14 (26.42)		
pCC	14 (26.42)		
ICC	13 (24.53)		
Gall bladder cancer	5 (9.43)		
Others	5 (9.43)^a^		
Previous chemotherapy		> 0.05	> 0.05
Yes	18 (33.96)		
No	34 (64.15)		
Unknown	1 (1.89)		
Splenomegaly		> 0.05	> 0.05
Yes	15 (28.30)		
No	38 (71.70)		
Steatosis		> 0.05	> 0.05
Yes	19 (35.85)		
No	29 (54.72)		
Unknown	5 (9.43)		
Fibrosis		> 0.05	> 0.05
Yes	16 (30.19)		
No	25 (47.17)		
Unknown	12 (22.64)		
Cirrhosis		> 0.05	> 0.05
Yes	2 (3.77)		
No	51 (96.23)		
BSA [mean ± SD]	2.0 ± 0.2	> 0.05	> 0.05
FRLV baseline, cm^3^ [mean ± SD]	357.0 ± 160.0	> 0.05	> 0.05
sFRLV baseline, % [mean ± SD]	20.8 ± 7.7%	> 0.05	> 0.05
Segment IV portal vein embolization		> 0.05	> 0.05
Yes	9 (16.98)		
No	44 (83.02)		
Hepatic vein embolization		> 0.05	> 0.05
Yes	19 (35.8)		
No	34 (64.2)		
Time after PVE to CT (days ± SD)	19.4 ± 6.4	0.033	> 0.05
Bilirubin mean ± SD (mg/dL), baseline	0.9 ± 1.1	> 0.05	> 0.05
GOT mean ± SD (U/L), baseline	59.7 ± 47.9	> 0.05	> 0.05
GPT mean ± SD (U/L), baseline	66.0 ± 63.9	> 0.05	> 0.05
GGT mean ± SD (U/L), baseline	372.0 ± 384.3	> 0.05	> 0.05

Data are expressed as *n* (%) unless otherwise specified

*HCC* Hepatocellular carcinoma, *CrC* Colorectal cancer, *ICC* Intrahepatic cholangiocarcinoma, *pCC* Perihilar cholangiocarcinoma, *SD* Standard deviation, *PVE* Portal vein embolization, *FRLV* Future remnant liver volume, *sFRLV* Standardized future remnant liver volume, *BSA* Body surface area, *CT* Computed tomography, *GGT* Gamma-glutamyl transferase, *GPT* Glutamic pyruvic transaminase, *GOT* Glutamic oxaloacetic transaminase, *Ig* Immunoglobulin

^a^Non-seminomatous germ cell tumors (*n* = 1), uveal malignant melanoma (*n* = 1), solitary fibrous tumor (*n* = 1), liver adenoma (*n* = 1), IgG4-associated mass

^b^Linear logistic regression for sFRLV ratio (sFRLV post PVE/sFRLV baseline)

^c^Binary logistic regression for sFRLV hypertrophy ≥ 1.33

### Future Remnant Liver Hypertrophy After PVE

FRLV increased significantly from 357.0 ± 160.0 mL at baseline to 516.5 ± 185.1 mL after PVE (*p* < 0.001). sFRLV also increased significantly from 20.8 ± 7.7% at baseline to 30.4 ± 8.7% after PVE (*p* < 0.001), resulting in a mean hypertrophy of future remnant liver (sFRLV post/sFRLV baseline) by 1.5 ± 0.3-fold. There was no significant difference in FRL hypertrophy after PVE compared with combined PVE and HVE (1.5 ± 0.4 vs. 1.5 ± 0.3; *p* = 0.80). Moreover, there was also no significant difference in FRL hypertrophy after additional embolization of segment IV embolization compared with right-sided PVE only (1.6 ± 0.3 vs. 1.5 ± 0.4; *p* = 0.37). Linear regression showed that the number of days after PVE within the time range of 2–5 weeks until volumetry (*p* = 0.033), and female sex (*p* = 0.045), were positively associated with a higher FRL hypertrophy after PVE. All other baseline characteristics did not show any significant correlation with the observed FRLV hypertrophy ratio after PVE (Table 1).

To foster PVE response assessment and prediction, a dichotomous outcome parameter was defined as stated above: sufficient hypertrophy (increase of sFRL by ≥ 1.33) was reached in 35 (66%) patients, resulting in 18 (34%) patients remaining with a hypertrophy of < 1.33 after PVE. Of the 18 non-responders, 4 had received simultaneous PVE and HVE, of which only 1 patient proceeded to surgery in the long-term and did not show postoperative liver failure or mortality. Binary logistic regression did not identify any significant baseline characteristics (age, sex, height, weight, BMI, liver laboratory parameters, pre-existing liver disease, splenomegaly, prior chemotherapy, baseline volumetry, days after PVE to volumetry) to be predictive for achieving an sFRL hypertrophy of ≥ 1.33 (Table 1).

### Radiomics Analysis for Prediction of Future Remnant Liver Hypertrophy After PVE

Since no common baseline characteristics were able to predict response to PVE regarding hypertrophy of sFRLV, a radiomics analysis of baseline CT data was performed.

**Fig. 3:**
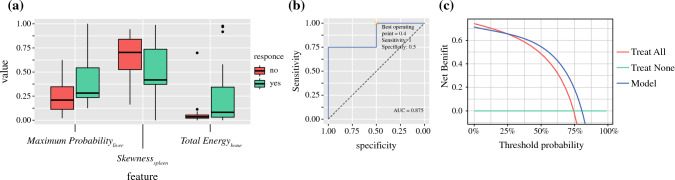
**a** Independent radiomic features of the baseline CT differentiated well between responders and non-responders to PVE. One feature each was identified for the liver, spleen and bone marrow. **b** ROC analysis of the resulting model based on all three radiomic features. Best operating point was set to a sensitivity of 1, resulting in a specificity of 0.5. **c** The decision curve shows that the radiomics-based model had a higher overall net benefit compared with scenarios in which no prediction model was used across the range of threshold probabilities (30–81%), considering a threshold probability of 66% for reaching an sFRLV hypertrophy of ≥ 1.33, as observed in our study cohort. *CT* Computed tomography, *PVE* Portal vein embolization, *sFRLV* Standardized future remnant liver

Three independent radiomic features (hereafter shown in italics) differentiated well between responders (sFRLV hypertrophy by ≥ 1.33) and non-responders (sFRLV hypertrophy by < 1.33), as shown in Fig. 3a.

- *MaximumProbability* of the liver (*MaximumProbability*_*liver*_), representing the occurrence of the most predominant pair of neighboring intensity values in baseline CT data related to the segmented liver volume.
- *Skewness* of the spleen (*Skewness*_spleen_), representing the asymmetry of the distribution of values about the mean value in baseline CT data related to the segmented spleen volume.
- *TotalEnergy* of the bone (*TotalEnergy*_*bone*_*)*, representing the magnitude of voxel values scaled by the volume of the voxel in baseline CT data related to the segmented first lumbar vertebra bone marrow.

Larger values of *MaximumProbability*_*liver*_ and *TotalEnergy*_*bone*_, and lower values of *Skewness*_spleen_ resulted in a higher probability of responding to PVE. Thus, one feature each per segmented region independently discriminated between responders and non-responders to PVE, as correlation analysis revealed (electronic supplementary Fig. 1a).

When assessing the predictive value of this model, the ROC analysis for the discrimination of responders and non-responders revealed accuracy with an AUC of 0.875 (Fig. 3b). Since PVE is an interventional procedure preceding major hepatectomy as mostly the only remaining curative option in patients with primary or secondary liver cancer, a model for sFRLV hypertrophy prediction must avoid the exclusion of patients from PVE who would have shown sufficient hypertrophy and who thus would have been eligible for surgery and curative treatment. Therefore, the best operating point of the model at the ROC was set to a sensitivity of 1, resulting in a best operating point at 0.4 (Fig. 3b), with a specificity of 0.5, a positive predictive value (PPV) of 0.769 and a negative predictive value (NPV) of 1.

The radiomics signature was built according to multivariate logistic regression analysis.

Here, the corresponding values for *MaximumProbality*_liver_, *Skewness*_spleen_, and *TotalEnergy*_bone_ were inserted for each patient of the validation dataset. The optimal model-derived cut-off (best operating point = 0.4) (Fig. 3b) was applied to the validation dataset to perform stratification for responding to PVE:

$$p \ge 0.4:{\text{predicted}}\;{\text{hypertrophy}}\;{\text{of}} \ge 1.33$$

$$p < 0.4:{\text{predicted}}\;{\text{hypertrophy}}\;{\text{of}} < 1.33\;{\text{or}}\;{\text{non-hypertrophy}}.$$

Decision curve analysis can assess the effectiveness of the created model regarding clinical utility by determining the net benefit. In addition, considering a threshold probability of 66% for reaching an sFRLV hypertrophy of ≥ 1.33, as observed in our study cohort, decision curve analysis showed that the radiomics-based model had a higher overall net benefit than the no-prediction model (i.e., treat all or treat none scheme) in predicting sFRLV hypertrophy across the range of threshold probabilities between 30 and 81%.

## Discussion

In this study, multi-organ radiomics analysis of baseline CT data is evaluated for prediction of future remnant liver hypertrophy after portal vein with or without HVE. The designed model included CT data of the liver, spleen, and bone, representing a thorough assessment of contributors to liver regeneration. First, analysis of liver parenchyma was included since it may be altered due to liver cirrhosis, prior chemotherapy, or other underlying disease, restricting its potential for hypertrophy. Although it is known that liver cirrhosis and prior chemotherapy are risk factors for postoperative FRL failure, meta-analysis^30^ of available studies evaluating liver cirrhosis^31–36^ and prior chemotherapy,^37–42^ as well as our data, did not identify these variables as independent predictors of hypertrophy after PVE, emphasizing the need for more elaborate assessment of baseline status prior to PVE. Second, imaging data of the spleen was added to the presented radiomics approach, since it is known that molecular factors regulated by the spleen affect liver cirrhosis and the potential of liver regeneration,^43,44^ and that splenic imaging parameters can support the prediction of liver-associated disease such as portal hypertension.^45,46^ Third, bone marrow imaging data were included in the radiomics model, since it is known that stem cells/bone marrow-derived liver sinusoidal endothelial cells (LSEC) contribute to liver regeneration,^47,48^ although the exact ways of mechanisms remain unknown,^49^ and bone marrow suppression hinders adequate liver regeneration after partial hepatectomy.^50^ Interestingly, our analysis identified each independent radiomic feature per organ (liver, spleen, bone marrow) to be predictive for FRL hypertrophy, potentially reflecting the addressed underlying mechanisms of liver hypertrophy and liver–spleen–bone marrow crosstalk. The herein presented model showed better sensitivity and specificity than reported for a radiomics model based on a small CT-guided region-of-interest analysis of liver parenchyma alone.^24^ To the best of our knowledge, this is the first study linking multi-organ radiomics imaging data with liver regeneration potential, emphasizing the importance of the liver–spleen–bone marrow axis. Future studies should further address which, and how, underlying molecular pathways and processes of these organs contribute to liver regeneration after PVE and how these findings may support understanding of liver tissue regeneration and hypertrophy prediction. This may also be further supported by the additional combination of potential predictive baseline characteristics and radiomic approaches.^25^

We showed that the derived radiomics model enables to discriminate between responders and non-responders to PVE with high sensitivity and moderate specificity. From a surgical point of view, a model that securely identifies non-responders and certainly avoids false negative prediction while having to accept potential false positive predictions of FRL hypertrophy is more useful than vice versa. This will ensure the most important goals of avoiding both (1) unnecessary liver preparation procedures such as PVE, and (2) falsely precluding patients from this (last) curative treatment option before entering palliative care. Our presented model shows the clinically meaningful NPV of 1. Moreover, we could show that the use of the presented radiomics approach has a positive net benefit and may thus improve clinical decision making regarding liver preparation techniques prior to major hepatectomy. However, our data would have to be confirmed in larger prospective studies prior to being implemented in clinical routine.

The study has some limitations. First, the patient cohort was small for radiomics studies, which preferably require large training datasets to optimize prediction models. Further studies with larger multicentric cohorts for external validation are needed to generalize the results presented here. This feasibility study is meant to generate first evidence supporting the design of such studies as well as to guide basic research aiming to find molecular mechanisms involved in liver hypertrophy.

Second, the underlying imaging data are derived from a heterogenous patient cohort regarding the underlying primary or secondary liver cancer as well as varying CT scanner hardware. However, considering potential future clinical implementation of radiomics models, algorithms will need to be capable of handling multicentric imaging data of various vendors. Furthermore, this study included patients receiving PVE with or without simultaneous HVE, since no difference in hypertrophy was found in our data as well as in recent studies.^19,51^ However, other studies reported a superior hypertrophy after simultaneous embolization of portal and hepatic veins,^52,53^ which may be partly influenced by lower baseline FRLV in the combined group compared with the group receiving PVE only in these studies (a parameter known to be negatively correlated with hypertrophy).^30,54,55^ However, simultaneous PVE and HVE seems superior regarding faster hypertrophy and increase in FRL function,^53^ both of which were not within the scope of this study, but not necessarily to achieve final FRLV hypertrophy evaluated here. While we could show that additional HVE or other baseline data did not contribute to bias in our radiomic analysis, these factors should be considered in future studies. In this context, any liver preparatory procedure, regardless of the technique used, would benefit from improved prospective hypertrophy prediction, as aimed for in this feasibility study.

Third, the desirable outcome of preparatory procedures prior to hepatectomy, such as PVE, is still a matter of debate in the current literature, and thus the prediction of volume hypertrophy as performed here may not be fully applicable in clinical practice. In this context, although also in discussion, an sFRLV of at least 20–25%, 30%, or 40% is commonly used by clinicians as a minimum volume of FRL for patients with a healthy liver, prior chemotherapy, or liver cirrhosis.^5,23,30^ Since this feasibility study aimed to predict actual liver volume hypertrophy (which a patient with an increased sFRLV, for example from 29.5 to > 30%, and thereby reaching sFRLV cut-off analysis, would not have), which is best reflected by a degree of hypertrophy ratio analysis as performed here, an arbitrary FRLV hypertrophy of ≥ 1.33 was chosen as the response parameter for this study and was thus similarly designed as comparable studies.^24,25^ However, future studies should also consider other endpoints such as successful transfer to surgery, which may be confounded by other parameters such as metastatic spread to FRL or tumor growth to inoperability. Nonetheless, although it is known that FRLV is a major predictor of postoperative liver failure,^3,4^ recent studies have shown that assessment of FRL function, instead of volume, by hepatobiliary scintigraphy or gadoxetic acid-enhanced magnetic resonance imaging (MRI) might be valuable to evaluate eligibility for surgery or predict postoperative liver failure. Importantly, the radiomic-based methods used in this study for prediction of PVE response can be transferred to imaging techniques such as MRI.^13,15,56–59^ In this context, gadoxetic acid-enhanced MRI might be a promising one-stop-shop modality for assessing FRL volume and function as well as for predicting hypertrophy after liver preparatory procedures prior to hepatectomy.^60^

## Conclusions

This proof-of-concept study provides first evidence regarding the importance of the liver–spleen–bone marrow axis, as investigated by radiomic analysis of baseline CT data for prediction of future remnant liver hypertrophy after PVE prior to major hepatectomy in primary and secondary liver cancer.

### Supplementary Information

Below is the link to the electronic supplementary material.

Supplementary Fig. 1. In case of several relevant features per response criterion, a correlation matrix was calculated. (a) Correlogram including independent radiomic features where clusters of textural features became apparent. These indicate a strong correlation between parameters of the same imaging method. Blue circles indicate positive correlation, red circles indicate negative correlation. (b) Summary of decision curve analysis characteristics shown in Fig. 3c. (TIF 866 KB)
