# Supplementary figures and images for: Multi-organ Radiomics-Based Prediction of Future Remnant Liver Hypertrophy Following Portal Vein Embolization
Source: Ann Surg Oncol. 2023 Sep 5;30(13):7976–85. doi: 10.1245/s10434-023-14241-5 (PMC10625940; doi:10.1245/s10434-023-14241-5)

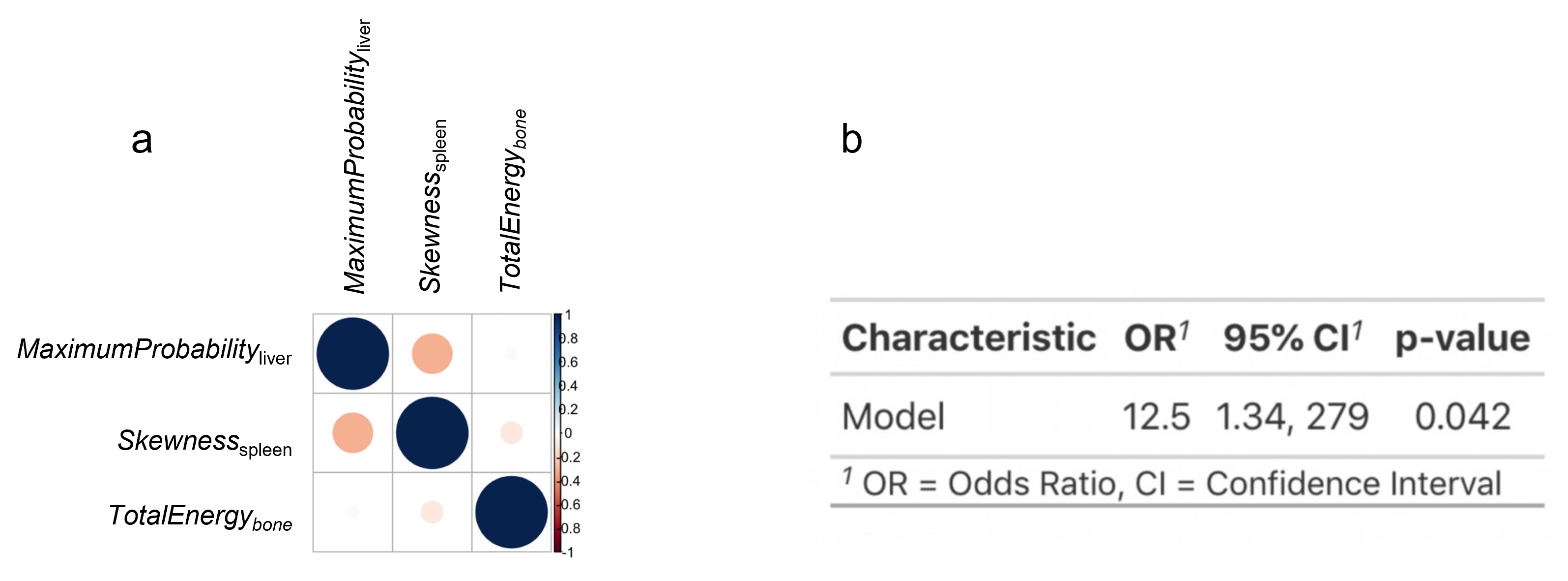

Supplement: Supplementary file 1 — Supplementary Fig. 1. In case of several relevant features per response criterion, a correlation matrix was calculated. (a) Correlogram including independent radiomic features where clusters of textural features became apparent. These indicate a strong correlation between parameters of the same imaging method. Blue circles indicate positive correlation, red circles indicate negative correlation. (b) Summary of decision curve analysis characteristics shown in Fig. 3c. (TIF 866 KB) [file 10434_2023_14241_MOESM1_ESM.tif]
